# Native Wound-Repair Proteins Retained in Multilayer Placental CAMPs

**DOI:** 10.3390/ijms262010121

**Published:** 2025-10-17

**Authors:** Pragya Singh, Shantanu Guha, Odalis Landa, Andrew Ryan King, Diego Valdes Cavazos, Joanna Marquez, Shauna Hill

**Affiliations:** RegenTX Labs LLC, 3463 Magic Dr Ste 315, San Antonio, TX 78229, USA

**Keywords:** CAMPs, full-thickness, ACA, wound covering, human placenta, placental allografts, global proteomics

## Abstract

The human placenta is a complex organ that supports fetal development and is rich in extracellular matrix proteins and growth factors, making it suitable as a biomaterial in wound care. Placenta-derived amnion-only allografts have traditionally been used in the clinic, but they lack the structural and biochemical complexity of the full three-layer placental membrane, which includes the amnion, intermediate, and chorion layers. Advances in tissue engineering have enabled preservation of multiple layers, giving rise to multilayer placental-based Cellular and Acellular Matrix-like Products (CAMPs) such as Full-Thickness (FT; amnion, intermediate, chorion) and ACA (amnion, intermediate, chorion, amnion). Although these advanced CAMPs are increasingly applied clinically, their molecular composition has not been comprehensively defined. This study presents a global proteomic analysis of FT and ACA, complemented by targeted multiplex analysis of soluble proteins and an in vitro angiogenesis assay. Proteomic profiling identified 8908 structural and bioactive components, with 32.5% of proteins associated with tissue repair and remodeling pathways. Multiplex analysis confirmed accessibility of biologically relevant soluble factors. Endothelial tube formation assays further supported biological relevance, demonstrating that soluble proteins in FT and ACA support angiogenesis. These data provide a molecular characterization of multilayer CAMPs and underscore their potential to deliver durable wound coverage while supporting the local microenvironment.

## 1. Introduction

The human placenta is a complex organ that has long been utilized in medicine as a biomaterial [1]. Its native function is to act as a protective barrier while facilitating nutrient and gas exchange. Its composition, which includes extracellular matrix (ECM) proteins, growth factors, and other bioactive molecules, offers structural and biological properties that make it valuable as a biomaterial [2,3,4]. One of its most common medical applications is in wound care, where its inherent barrier function is leveraged to serve as a wound covering, protecting the wound, reducing the risk of contamination, and maintaining a local environment that supports tissue repair. In clinical practice, placental membranes are engineered into allografts that retain these properties and are broadly categorized as Cellular and Acellular Matrix-like Products (CAMPs), which are routinely applied as wound coverings in modern medical practice [5,6].

Placental allografts have traditionally been engineered as amnion-only membranes. Amnion is the thinnest layer of the placenta, composed of a collagen-rich matrix with well-documented anti-inflammatory and epithelial-supportive properties [7,8]. Despite its clinical utility, amnion alone lacks the full structural and biochemical complexity of the native placental membrane. In addition to amnion, the placenta contains an intermediate layer and chorion, which contribute distinct biological features. The intermediate layer is a rich source of soluble biomolecules, with over 900 identified to date [9], while the chorion is the thickest layer and is enriched in ECM proteins such as collagen, fibronectin, laminin, and elastin [10,11]. Recent advances in tissue engineering have enabled the retention of all three layers, resulting in allografts that are both structurally more robust and compositionally more diverse. These advances have laid the groundwork for the development of next-generation CAMPs that better preserve the native characteristics of the placenta.

Building on these advances, multilayer CAMPs such as Full-Thickness (FT; amnion, intermediate, chorion) and ACA (amnion, intermediate, chorion, amnion) represent the next stage in placental allograft development. Previous studies have demonstrated that these CAMPs retain higher levels of extracellular matrix proteins and growth factors including ANG-2 (angiopoietin-2), EGF (epidermal growth factor), PDGF-AA (platelet-derived growth factor), and VEGF (vascular endothelial growth factor), as well as signaling factors associated with angiogenesis, tissue remodeling, inflammation, and host defense [12,13,14,15]. By preserving the full membrane structure, these CAMPs may improve graft durability and establish a microenvironment more favorable to tissue repair [16,17]. 

Previous studies have relied on targeted assays that capture only a limited subset of proteins or focus on individual tissue layers, leaving the broader proteomic landscape of these advanced CAMPs unexplored. This lack of comprehensive characterization restricts our understanding of how retention of the full membrane architecture contributes to graft composition and biological relevance.

To address this gap, the present study provides a molecular analysis of two advanced CAMP technologies, FT and ACA, in their ready-to-use form. We combined untargeted global proteomic profiling, targeted multiplex analysis of soluble proteins, and an in vitro angiogenesis assay to evaluate both molecular composition and associated biological relevance. Together, these findings offer new insights into the structural and compositional integrity of advanced CAMPs and highlight their potential to provide durable wound coverage while supporting a local environment conducive to tissue repair.

## 2. Results and Discussion

### 2.1. Proteomic Composition of Multilayer Placental Technologies

Two multilayer CAMP technologies were evaluated for their structural characteristics and overall protein composition in their ready-to-use form. The Full-Thickness (FT) technology retains the three native placental layers amnion, intermediate, and chorion. The four-layer ACA technology contain these same layers together with an additional preserved second amnion layer.

**Figure A1:**
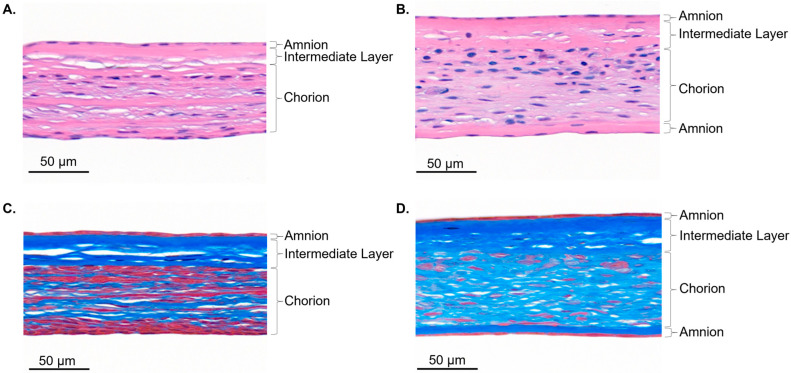
Histological Evaluation of FT and ACA Technologies. (**A**,**B**) Representative tissue cross-sections of FT (**A**) and ACA (**B**) constructs with H&E staining showcasing structural configuration. (**C**,**D**) MTS staining for FT (**C**) and ACA (**D**) illustrates the distribution of collagen content. Scale bar = 50 µm. The representation of the histological cross-sections is of at least eight donors per CAMP technology.

Histological analysis demonstrated the presence and organization of these membrane layers in both technologies (Appendix A Figure A1). Hematoxylin and Eosin (H&E) staining demonstrated preservation of membrane architecture, and Masson’s Trichrome staining revealed dense, collagen-rich extracellular matrix (ECM) across both technologies (Appendix A Figure A1). These findings demonstrate the expected structural composition and support previous observations of increased collagen staining.

Building on this structural evaluation, proteomic profiling was performed on allografts derived from different donors. Previous global proteomic studies have characterized unprocessed placental tissue [18], providing insights into the native protein composition. Extending this type of analysis to processed placental allografts, this study applied a global proteomic approach to multilayer CAMPs in their ready-to-use form.

**Figure 1:**
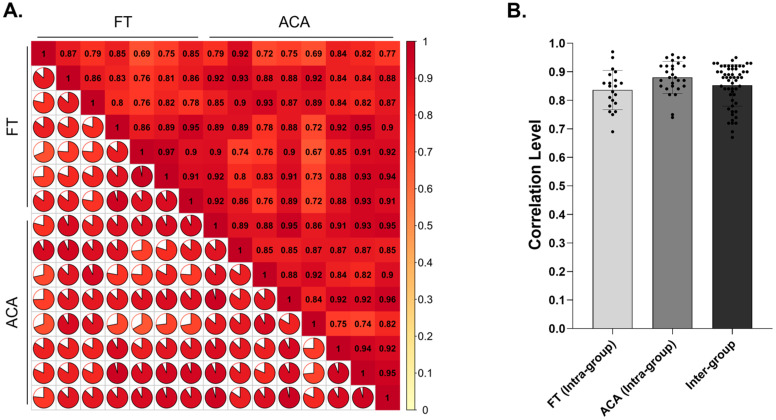
FT and ACA Technologies Demonstrate Similar Global Protein Composition. (**A**) The Pearson’s correlation coefficient (R) was used to assess the correlation of biological replicates based on the overall expression of proteins identified between FT and ACA. The correlation heatmap visualizes intra- and inter-group relationships, where the closer to |R| = 1, a stronger the correlation between samples. (**B**) The correlation of FT and ACA with respect to intra- and inter-group. Correlation analyses were quantified in seven distinct donors from FT and eight from ACA technology. Error bars represent the ±SD.

A total of 8908 proteins were detected as shared between FT and ACA. Protein abundance profiles were consistent across samples, with Pearson correlation coefficients of 0.84 within FT, 0.88 within ACA, and 0.85 between groups (Figure 1A,B). In Figure 1A, a modified heat map is shown to visually demonstrate the correlation between all samples and technologies tested. The plot is divided into two analogous sections across the diagonal axis, representing intra-group (ACA vs. ACA; FT vs. FT) and inter-group (ACA vs. FT) comparisons. The top half displays Pearson correlation coefficients as numerical values, color-coded according to the similarity scale (0 = complete dissimilarity; 1 = exact match). The diagonal axis represents self-comparisons, with coefficients of 1.0 indicating 100% alignment. The bottom half shows corresponding pie charts that represent the percentage of overlap between each sample comparison. In Figure 1B, these correlation coefficients (excluding self-comparisons) are summarized in a bar graph, highlighting the distribution of intra-group (ACA vs. ACA; FT vs. FT) and inter-group (ACA vs. FT) comparisons. These results demonstrate that both CAMPs exhibit a similar proteomic profile, indicating a high degree of shared proteins.

**Figure A2:**
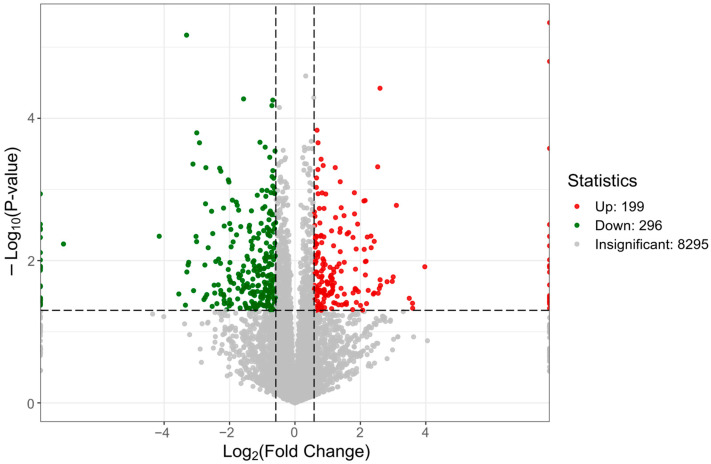
Total Proteome Volcano Plot Comparing ACA to FT. Colored data points represent proteins statistically significant differences between ACA and FT. Red points indicate proteins significantly upregulated in ACA, while green points indicate proteins significantly downregulated relative to FT.

The proteomic profile of ACA is largely shared with that of FT. Across the proteome, the relative levels of proteins are highly similar between the technologies, which is expected given that both technologies retain the same membrane layer types, with the ACA technology preserving an additional amnion layer. This is further illustrated in the volcano plot comparing ACA to FT (Appendix A Figure A2). With FT protein identification and relative expression as reference, approximately 5.6% of proteins in ACA are differentially expressed. These results confirm that FT and ACA share similar proteomic profiles.

**Figure 2:**
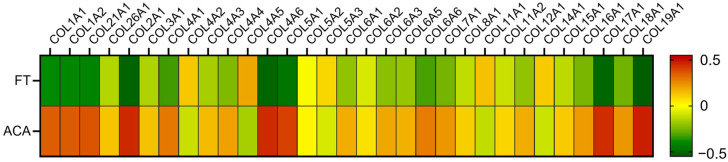
Relative Expression of Detected Collagen Isoforms. The relative expression of 31 collagen isoforms visualized as a heatmap with FT (seven distinct donors) and ACA (eight distinct donors) technologies. The color scale (−0.5 to 0.5) is used to indicate relative expression where red tones designate a higher relative enrichment, and green tones designate a lower relative enrichment.

Previous studies have shown that FT and ACA retain similar levels of selected growth factors and ECM-associated proteins, with collagen being the primary differentiating factor [13,14]. These findings are further supported by the current dataset, which confirms up to 50%, or 0.5-fold, higher levels of 31 collagen isoforms in ACA compared to FT (Figure 2). The higher relative levels of collagen isoforms are attributed from the preservation of an additional amnion layer, which is naturally rich in collagens [14]. This study identified elevated levels of Collagens I, II, III, IV, V, VI, XVII, XVIII, and XVIV in ACA compared to FT technologies. These results align with previous reports showing that ACA allografts contain significantly higher total collagen than FT allografts [14]. Higher collagen content may enhance the mechanical stability and handling characteristics of ACA in clinical use, given its influence on the structural properties of CAMPs [9,13,19].

### 2.2. Pathway Enrichment Analysis of the CAMP Proteome

To further characterize the CAMP proteome and assess its biological relevance, pathway enrichment analysis was performed on the 8908 detected proteins in both FT and ACA. Proteins were annotated by Gene Ontology (GO) biological processes and cellular components to better understand their subcellular localization and functional roles. This analysis aimed to identify key cellular pathways and mechanisms supported by the CAMPs technology.

**Figure 3:**
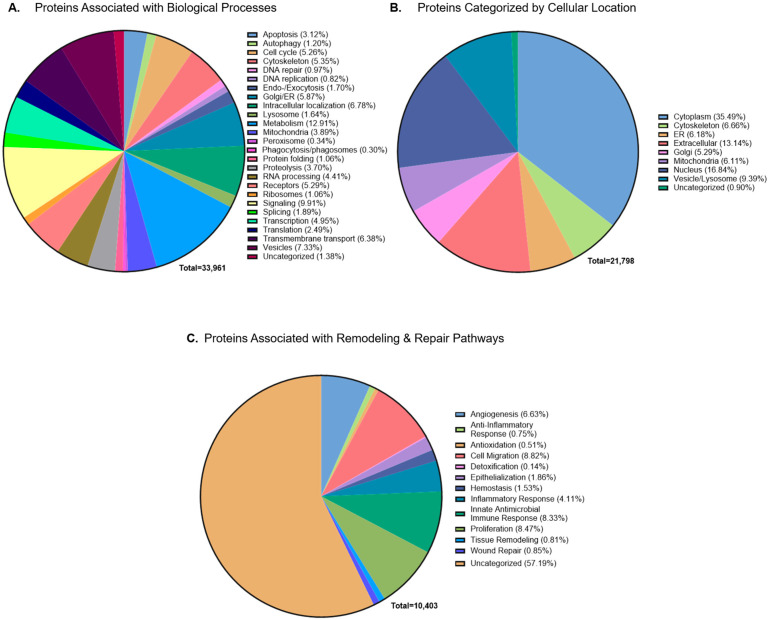
Classification of Global Proteomic Expression of FT and ACA Technology by Biological Processes, Cellular Localization, and Tissue Repair. Distribution of shared detected proteins classified by (**A**) high-level biological processes related to specific cellular pathways and structures, (**B**) cellular location, and (**C**) a custom GO process list related to tissue remodeling and repair. The ‘Total’ number to the bottom right of each pie-chart refers to the number of hits within all pathways specified.

Annotation resulted in 33,961 assignments across GO categories, with many proteins mapped to more than one biological process. Analysis of biological processes revealed that the most represented processes include metabolism, signal transduction, and intracellular transport (Figure 3A). These pathways are fundamental for supporting cell viability, regulating signaling and supporting molecular trafficking.

In addition to biological processes, the detected proteins were also mapped to cellular components to identify subcellular localization. This analysis resulted in 21,798 assignments across compartments, with the majority localized to the cytoplasm, followed by the nucleus and extracellular space (Figure 3B). This distribution indicates that CAMPs retain a broad spectrum of intracellular proteins as well as extracellular components, the latter being particularly relevant as extracellular proteins contribute to matrix remodeling and the regulation of the local cellular environment during tissue repair.

**Figure A3:**
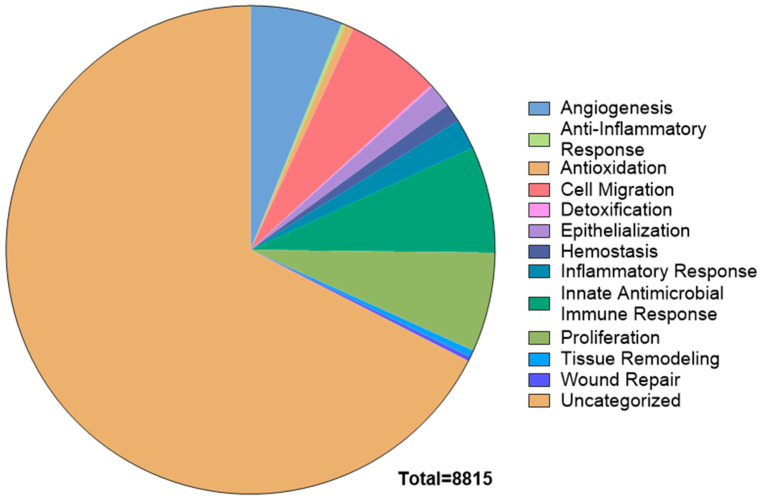
Classification of FT and ACA Proteomes into Repair Pathways Using Single-Pathway Assignment.

Building on this, the proteome was further evaluated to determine whether the retained proteins are linked to biological processes associated with tissue repair. This analysis generated 10,403 mapping assignments across twelve repair-associated categories, as individual proteins contribute to more than one process. The categories included angiogenesis, inflammation, epithelialization, proliferation, hemostasis, antioxidant activity, detoxification, antimicrobial activity, innate immune response, and general wound healing associated mechanisms were employed [20,21,22,23,24,25,26,27,28,29,30]. For this, a custom classification approach was applied to group proteins into twelve functional categories encompassing key repair-associated processes (Table 1). Proteins from both FT and ACA were represented across all twelve categories (Figure 3C). Using a conservative classification approach that restricted each protein to a single pathway, 32.52% of the shared proteome was associated with repair-associated processes (Appendix A Figure A3).

### 2.3. Evaluation of Soluble Proteins Under Physiological Conditions

To complement the global proteomic analysis, this study evaluated the availability of soluble proteins from both CAMP technologies following hydration. The objective was to determine which proteins may become accessible in the local environment under physiologically relevant conditions and whether these proteins are associated with biological processes involved in tissue repair. CAMPs were incubated at 37 °C for 72 h, and the resulting conditioned media containing solubilized proteins was analyzed using multiplex immunoassays targeting a broad panel of cytokines and growth factors. In total, 507 soluble analytes were detected across both CAMPs, with similar distribution patterns observed between groups.

**Figure 4:**
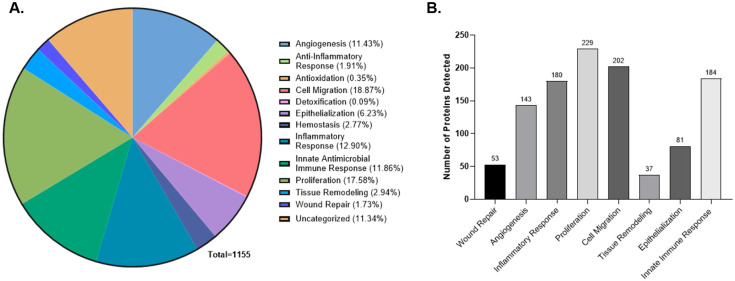
Protein Association Analysis of Soluble Components from FT and ACA. (**A**) All detected proteins in the FT and ACA groups were annotated and categorized using a custom GO pathway. list associated with biological processes relevant to tissue remodeling and repair. (**B**) The bar graphs represent the number of proteins detected in each category. Proteins associated with multiple biological processes were included in more than one category. The detected protein was annotated in four donors per FT and five donors per ACA technology.

To assess the potential biological relevance of these proteins, detected analytes were mapped to a predefined set of biological processes associated with tissue remodeling (Figure 4A). This classification, based on GO annotations, included key pathways such as proliferation (229 proteins), cell migration (202 proteins), and angiogenesis (143 proteins), as well as epithelialization, inflammatory response, and innate immune activity (Figure 4B). Importantly, the proteins detected in this study overlap with those reported in unprocessed placental tissue, further indicating that the molecular features of the native membrane are preserved in these technologies [32].

These findings are consistent with prior studies demonstrating that three-layer placental technologies retain growth factors and ECM-associated proteins detectable under similar in vitro conditions [31,33]. Together with the retained proteome data, these results offer a more complete molecular characterization of placental technologies. The presence of soluble proteins linked to biological processes associated with repair suggests that these CAMPs may help support a local environment favorable for tissue remodeling.

### 2.4. In Vitro Angiogenesis Assessment of Soluble Proteins

The global and targeted proteomic analysis showed that CAMPs retain proteins associated with wound repair. To demonstrate the functional relevance of available soluble proteins detected in the FT and ACA allografts, an in vitro tube formation assay was conducted to assess whether available proteins can support endothelial cell activity associated with angiogenesis. Angiogenesis represents a central process in tissue repair, as the formation of blood vessels enables delivery of oxygen and nutrients, removal of waste, and establishment of a biologically conducive microenvironment [20].

**Figure 5:**
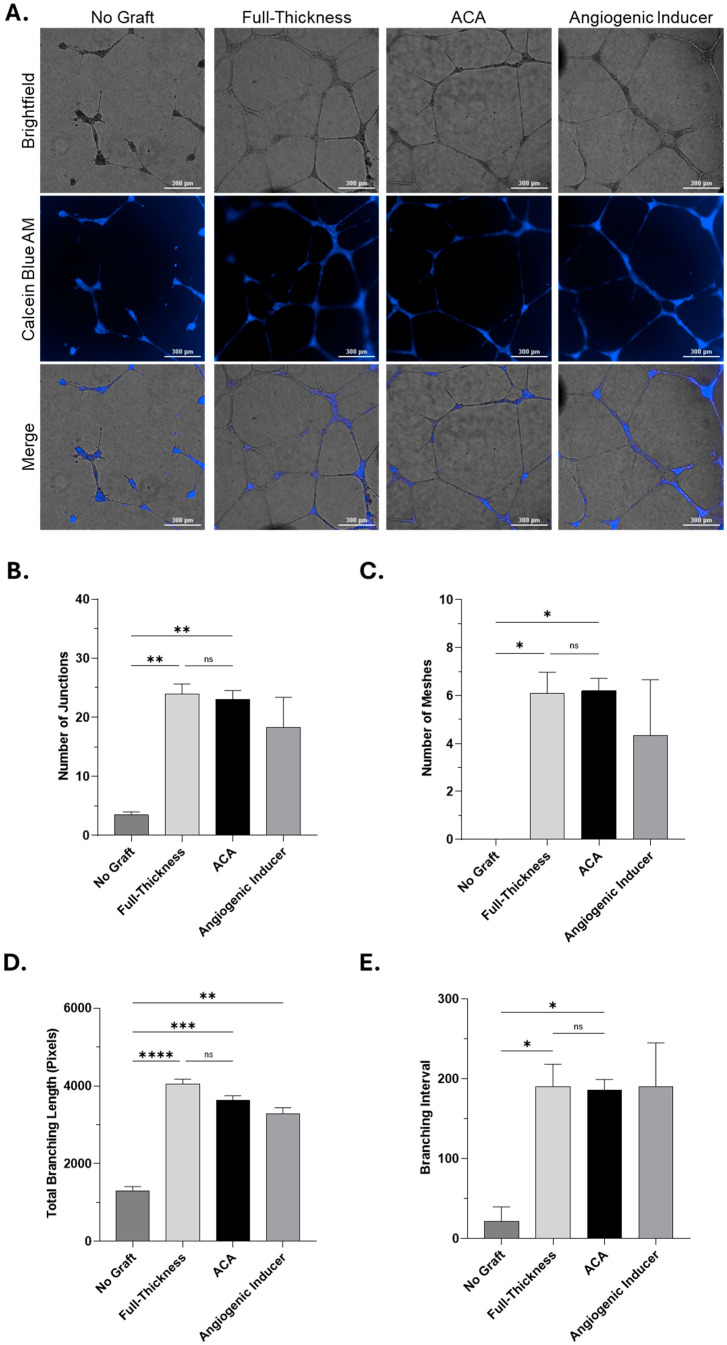
Endothelial Tube Formation with Soluble Protein from FT and ACA. Tube formation on HUVECs was evaluated after 16 h with no graft, soluble proteins from FT and ACA, or angiogenic factors. (**A**) Representative images indicate tube formation and network structure visualized via brightfield, Calcein Blue AM fluorescent staining, and merged overlays. Network-like tubular structures formed by soluble proteins from FT and ACA were comparable to those induced by angiogenic factors. The assay was conducted from four distinct donors from FT and seven distinct donors from ACA. The representative image is from one independent study. Scale bar = 300 µm. Bar graphs show quantification of (**B**) Number of Junctions, (**C**) Number of Meshes, (**D**) Total Branching Length, and (**E**) Branching interval using ImageJ Fiji Software (Java Version 21.0.7) plugin Angiogenesis Analyzer (1.0.C Version). Data is expressed as mean ± SEM. Analyzed by Gaussian one-way ANOVA (ns, not significant; *, *p* < 0.05; **, *p* < 0.01, ***, *p* = 0.0001, ****, *p* < 0.0001).

In this study, primary human umbilical vein endothelial cells (HUVECs) were cultured on Geltrex matrix and incubated with conditioned media collected from FT and ACA allografts after 72 h at 37 °C. Basal media alone served as a negative control, while media supplemented with an angiogenic inducer was used as a positive control. After 16 h of incubation, tube formation was qualitatively assessed using brightfield and fluorescence imaging using Calcein Blue AM. Compared to the negative control, conditioned media from both FT and ACA groups supported the formation of capillary-like structures (Figure 5). These included elongated tubes, branching points, and enclosed meshes similar to those observed in the positive control, indicating that proteins available in the media can contribute to a local environment that supports endothelial network formation.

Quantitative analysis further confirmed these observations, demonstrating higher numbers of junctions (Figure 5B), meshes (Figure 5C), and overall branching (Figure 5D,E) in both FT and ACA groups relative to the negative control, with values comparable to the angiogenic inducer. Notably, no significant differences were observed between FT and ACA allografts, indicating that both technologies provide a comparable capacity to support endothelial network formation.

These findings align with previous literature evaluating placenta-derived materials and their ability to support neovascularization in vitro [34,35,36,37]. Importantly, they provide functional context to the proteomic and multiplex findings presented earlier. While molecular analyses confirmed the presence of growth factors associated with angiogenic pathways, these results demonstrate that the proteins remain in a state that can provide a conducive environment to support tissue repair and remodeling under relevant in vitro conditions.

## 3. Materials and Methods

### 3.1. Placental Membrane-Derived CAMP Technologies

The placental tissue, donated from consenting healthy birth mothers over the age of 18 following cesarian and vaginal deliveries, was screened and tested before processing in accordance with U.S. Food and Drug Administration (FDA) regulations and American Association of Tissue Banks (AATB) standards. Placental based allografts were generated as described in [14]. Briefly, FT is a three-layer allograft that consists of amnion, intermediate, and chorion layers, while ACA is a more advanced four-layer allograft that includes FT layers with an additional amnion layer preserved during processing. Placental allografts used in this study include Full-Thickness technology (CompleteFT™, Tiger Wound Care LLC, Conshohocken, PA, USA) and ACA technology (ACApatch™, Tiger Wound Care LLC, Conshohocken, PA, USA).

### 3.2. Histological Assessment

Histology analysis of FT and ACA technology was conducted utilizing Hematoxylin and Eosin (H&E) and Masson’s trichrome staining. For the staining, samples were fixed overnight utilizing 10% neutral-buffer formalin (VWR, Radnor, PA, USA), 5 µm-thick cross-sections of the allografts were utilized, and stained by the Precision Pathology Laboratory (San Antonio, TX, USA) according to their standardized protocols. H&E staining was utilized for the assessment of the differences in the tissue structures between ACA and Full-Thickness (FT) technology. MTS staining was used for the qualitative ECM collagen content assessment. The representative cross-section images of H&E and Masson’s trichrome staining were taken using Slide Viewer (Version 2.8.0) at a 60× magnification. For histological analyses, eight distinct donors from FT and ten from ACA were evaluated.

### 3.3. Global Proteomic Profiling

Sample preparation and LC-MS/MS acquisition for global proteomic analysis were conducted by Metware Biotechnology Inc. (Woburn, MA, USA). Full-Thickness (*n* = 7 donors) and ACA (*n* = 8 donors) were cryopulverized in liquid nitrogen and processed via ethanol solution precipitation, followed by lysis with 8 M urea, 1 mM PMSF, and 2 mM EDTA lysis buffer. The protein concentration was determined using a BCA assay kit (Thermo Scientific, Waltham, MA, USA). Proteins were subsequently separated based on hydrophobicity using a NanoElute UHPLC (Bruker, Billerica, MA, USA) system with a nanoliter flow rate on a C18 column for LC-MS/MS analysis. The mass spectrometry data were acquired using the ddaPASEF mode of a timsTOF HT mass spectrometer to establish an appropriate acquisition window for the diaPASEF acquisition method. The parameters used in the analysis included a valid gradient of 47 min, positive ion detection mode, parent ion scanning range of 100–1700 *m*/*z*, ion mobility 1/K_0 in the range of 0.6–1.6 Vs/cm^2^, ion accumulation and release time of 100 ms, ion utilization rate approximating 100%, capillary voltage of 1600 V, drying gas rate of 3 L/min, and drying temperature of 180 °C. Parameters used in the diaPASEF acquisition mode included a mass range of approximately 400–1200, a mobility range of 0.6–1.6 Vs/cm^2^, a mass width of 25 Da, a mass overlap of 0.1, 24 mass steps per cycle, and two mobility windows, totaling 48 acquisition windows.

#### 3.3.1. Proteomic Analysis

In database searching with DIA-NN (v1.8.1), precursor ion and protein-level identifications were filtered at a 1% false discovery rate (FDR), ensuring high confidence in peptide and protein assignments. For protein quantification, DIA-NN’s MaxLFQ algorithm was applied with normalization across samples to minimize systematic bias. Differential expression analysis was then performed using either t-tests (for two-group comparisons) or ANOVA (for multi-group comparisons) in FT and ACA replicates. For differential expression analyses which include *p*-value testing, FDR is calculated using the Benjamini–Hochberg (BH) method. Global protein expression profiles for FT and ACA were used to generate a custom heatmap for all collagen variants. Protein expression was determined by averaging protein expression across FT and ACA groups and assessing relative abundance of each variant to the overall average. Variance in protein abundance within each group was assessed by an ANOVA test, and associated *p*-values were calculated using unpaired two-tailed t-tests with Welch’s correction for the volcano plot.

#### 3.3.2. Bioinformatics and Functional Category Analysis

To characterize the biological relevance of total proteins within the global proteomics dataset, a comprehensive bioinformatics pipeline was applied. Functional enrichment analysis included Gene Ontology (GO) categories, Kyoto Encyclopedia of Genes and Genomes (KEGG) pathways, EuKaryotic Orthologous Groups (KOG) functional classification, subcellular localization, and signal peptide (SignalP) prediction. Functional categorization of proteins was performed using the GO-based clustering tool Categorizer [38], enabling the classification of detected proteins in FT and ACA technology into biologically relevant pathways. Gene Ontology (GO) terms encompassing biological pathways associated with tissue remodeling were curated from QuickGO (European Bioinformatics Institute, Hinxton, United Kingdom) [39] as listed in Table 1. This included over 170 GO terms mapped to the processes: wound repair, proliferation, cell migration, innate immune response, angiogenesis, inflammatory response, tissue remodeling, and epithelialization. These curated GO pathways were matched against the Homo sapiens annotation dataset from the EBI Gene Ontology Annotation (GOA) database [40] then mapped to the detected protein list derived from the global proteomic profiling to assign proteins for functional overlap where annotations intersected.

Downstream enrichment analyses (GO, KEGG, KOG, protein domains) were also evaluated with respect to significance *p*-values derived from hypergeometric testing against the background of all identified proteins. The raw *p*-values were further subjected to FDR correction through the BH method to control for multiple comparisons.

### 3.4. L-Series Human Antibody Array for Soluble Protein Profiling

The L-Series multiplex enzyme-linked immunosorbent assay (ELISA) array (RayBiotech, Inc., Peachtree Corners, GA, USA, Cat. No.: AAH-BLG-1-4) was used to semi-quantitatively assess 507 growth factors, cytokines, and other signaling molecules in the soluble extracts from FT and ACA. Soluble extract preparation used a surface area of 3 cm^2^ per 500 μL ratio of DMEM cell culture media for each placental allograft. Extracts were incubated at 37 °C at 700 RPM on a thermomixer (Eppendorf, Hamburg, Germany, Cat. No. 5382) for 3 days. After incubation, extracts were centrifuged at 10,000 RPM for 5 min to remove debris. The supernatant was sterilized using a 0.22 µm filter (VWR, Radnor, PA, USA, Cat. No. 29442-752). The samples were measured in duplicate and reported as Relative Fluorescence Units (RFUs). For the assay, extracts from four distinct FT donors and five distinct ACA donors were utilized.

The pathway-based bioinformatics approach used in the global proteomic dataset was applied to the L-Series antibody array dataset. Detected soluble analytes (*n* = 507) were compared against a curated list of GO biological pathways (Table 1). Proteins matching these pathways were further categorized using the AmiGO2 database to identify functional enrichment, as listed in Appendix A Table A1.

### 3.5. In Vitro Angiogenesis Assay

Angiogenic potential was assessed using the Angiogenesis Starter Kit Assay (Thermo Scientific, Waltham, MA, USA, Cat. No. A14609-01) following manufacturer’s instructions. Primary Human Umbilical Vein Endothelial Cells (HUVECs) (Thermo Scientific, Waltham, MA, USA, Cat. No. C0035C) were cultured in Medium 200 (Thermo Scientific, Waltham, MA, USA, Cat. No. M200500) supplemented with LVES (Thermo Scientific, Waltham, MA, USA, Cat. No A1460801) and maintained at 37 °C with 5% CO^2^. Cells were seeded at a density of 25,000 cells per well on Geltrex-coated 48-well plates (Thermo Scientific, Waltham, MA, USA, Cat. No. A1413202).

Experimental groups were treated with FT or ACA soluble extracts prepared as described in L-series Human Antibody Array with Medium 200 containing 2% heat-inactivated fetal bovine serum (VWR, Radnor, PA, USA, Cat. No.: 45000-734). Positive controls received growth media with LVES (containing angiogenic factors); negative controls received Medium 200 without LVES. Following a 16 h incubation, wells were washed with DPBS (Corning, Corning, NY, USA, Cat. No. 21-030-CV), and stained with Calcein Blue AM (6 µg/mL; Thermo Scientific, Waltham, MA, USA, Cat. No.: C1429) for 30 min at 37 °C. After a final DBPS wash, cells were imaged in brightfield and fluorescence modes using a BioTek Cytation 5 Multimode Reader (Agilent Technologies, Santa Clara, CA, USA) at 10× magnification.

Assays were performed using extracts from FT (*n* = 4 donors) and ACA (*n* = 9 donors) allografts, with three technical replicates per condition across two independent experiments.

#### Quantitative Analysis of Angiogenic Activity

Captured images were analyzed through NIH’s ImageJ (National Institutes of Health, Bethesda, MD, USA) with the Angiogenesis Analyzer plugin [41] to quantify the number of junctions, meshes, and branch lengths. Quantitative analysis was conducted in batches across three technical replicates of brightfield images per condition at 10× magnification after a 24 h treatment, accounting for non-selective bias. The following parameters were used in the Angiogenesis Analyzer plugin: minimum object size = 20 pixels, minimum branch size = 25 pixels, artificial loop size = 850 pixels, isolated element size threshold = 15 pixels, master segment size threshold = 15 pixels, and iteration number = 3 pixels. Statistical significance was determined using a normal Gaussian one-way ANOVA test using multiple comparisons of column means.

### 3.6. Data Analysis and Handling

For all analyses, a significance threshold of *p* < 0.05 was used. Statistical processing was conducted using GraphPad Prism (Version 10.5) and Microsoft Excel. Experiment specific statistical analyses are described in the corresponding methods subsections. To ensure reproducibility and independence of the data, all global and targeted proteomic analyses were performed by an independent third party, minimizing the potential for author-related bias. Analyses were conducted on biological replicates to confirm consistency across samples. Quantitative assessment of angiogenesis was conducted on representative experiments using ImageJ Fiji Software (Java Version 21.0.7) plugin Angiogenesis Analyzer (Version 1.0.C). This software applies objective algorithms for tube formation metrics and reduces the risk of subjective interpretation.

## 4. Conclusions

This study presents a detailed molecular characterization of advanced multilayer CAMPs. Through a combination of global proteomics, targeted multiplex protein detection, and an in vitro angiogenesis assay, we evaluated the protein composition and its relevance to tissue repair processes. The proteomic analysis revealed a highly consistent protein profile across both technologies, supporting the idea that the structural layers preserved in these CAMPs retain a diverse set of biologically relevant proteins. Functional categorization highlighted a substantial portion of these proteins associated with key processes involved in tissue remodeling and repair. Collagen was notably higher in ACA compared to FT, a distinguishing characteristic that aligns with previous studies and reflects the additional preserved amnion layer in the four-layer technology.

In addition to overall protein composition, the availability of soluble proteins was assessed. Soluble proteins detected in conditioned media were mapped to pathways associated with tissue repair, offering additional insight into the molecular profile accessible in ready-to-use CAMPs. An in vitro angiogenesis assay using endothelial cells further demonstrated that both FT and ACA technologies contain soluble proteins that can support endothelial tube formation. These findings demonstrate that the proteins preserve their functional integrity and provide a conducive environment to support cellular activities related to tissue repair.

Together, these findings demonstrate that CAMPs retain proteins linked to angiogenesis, cell proliferation, and immune modulation, which may contribute to a local environment supportive of tissue repair. This work advances the field by moving beyond targeted assays to offer broader proteomic profile of advanced CAMPs in their ready-to-use form. Preservation of these proteins may help retain the inherent biological properties of the tissue and provide a foundation for understanding the composition of placenta-derived CAMPs.

## 5. Patents

The subject of this manuscript is pending a patent and the IP rights are owned by Tiger Wound Care Medical, LLC, Conshohocken, PA, USA.

## Figures and Tables

**Table 1:** GO Pathways Selected for Custom Categorizer in Wound Repair Mechanisms. This table outlines the specific biological processes selected for the custom Categorizer list to evaluate the protein classification in FT and ACA. The left column indicates the biological processes, and the Gene Ontology (GO) pathway identifier and pathway description are in the right column. Each pathway selected was assessed by the researchers of this study for relevant parent and child nodes and branches to encompass the highest level of coverage for the mechanism specified in the left column.

Mechanisms	GO Pathways [Pathway Identifier (Description)]
Wound Repair	GO:0042060 (wound healing); GO:1903689 (regulation of wound healing, spreading of epidermal cells); GO:0035313 (wound healing, spreading of epidermal cells); GO:0061045 (negative regulation of wound healing); GO:0090303 (positive regulation of wound healing)
Angiogenesis	GO:0001525 (angiogenesis); GO:0048646 (anatomical structure formation involved in morphogenesis); GO:0048514 (blood vessel morphogenesis); GO:0001568 (blood vessel development); GO:0001944 (vasculature development); GO:0035239 (tube morphogenesis); GO:0035295 (tube development); GO:0060055 (angiogenesis involved in wound healing); GO:0061042 (vascular wound healing); GO:0061043 (regulation of vascular wound healing); GO:0002040 (sprouting angiogenesis); GO:1903672 (positive regulation of sprouting angiogenesis); GO:1903670 (regulation of sprouting angiogenesis); GO:1903671 (negative regulation of sprouting angiogenesis); GO:0001569 (branching involved in blood vessel morphogenesis); GO:1905553 (regulation of blood vessel branching); GO:1905554 (negative regulation of blood vessel branching); GO:1905555 (positive regulation of blood vessel branching); GO:0045766 (positive regulation of angiogenesis); GO:0016525 (negative regulation of angiogenesis); GO:0045765 (regulation of angiogenesis); GO:1903672 (positive regulation of sprouting angiogenesis); GO:1905555 (positive regulation of blood vessel branching); GO:0035470 (positive regulation of vascular wound healing)
Hemostasis	GO:0007599 (hemostasis); GO:0007596 (blood coagulation); GO:0072378 (blood coagulation, fibrin clot formation); GO:0072377 (blood coagulation, common pathway); GO:0030194 (positive regulation of blood coagulation); GO:1900047 (negative regulation of hemostasis); GO:1900046 (regulation of hemostasis); GO:1900048 (positive regulation of hemostasis); GO:0030195 (negative regulation of blood coagulation); GO:0071892 (thrombocyte activation); GO:0030193 (regulation of blood coagulation); GO:0030168 (platelet activation); GO:2000268 (positive regulation of blood coagulation, intrinsic pathway); GO:2000265 (positive regulation of blood coagulation, extrinsic pathway); GO:2000262 (positive regulation of blood coagulation, common pathway); GO:0002543 (activation of blood coagulation via clotting cascade)
Inflammatory Response	GO:0006954 (inflammatory response); GO:0090594 (inflammatory response to wounding); GO:0002544 (chronic inflammatory response); GO:0002526 (acute inflammatory response); GO:0002246 (wound healing involved in inflammatory response); GO:0002247 (clearance of damaged tissue involved in inflammatory response wound healing); GO:0106015 (negative regulation of inflammatory response to wounding); GO:0106016 (positive regulation of inflammatory response to wounding); GO:0106014 (regulation of inflammatory response to wounding); GO:1903034 (regulation of response to wounding); GO:0061041 (regulation of wound healing); GO:1903036 (positive regulation of response to wounding); GO:1903035 (negative regulation of response to wounding); GO:0009611 (response to wounding)
Proliferation	GO:0008283 (cell population proliferation); GO:0008284 (positive regulation of cell population proliferation); GO:0008285 (negative regulation of cell population proliferation); GO:0042127 (regulation of cell population proliferation); GO:0002043 (blood vessel endothelial cell proliferation involved in sprouting angiogenesis); GO:0048144 (fibroblast proliferation); GO:0048145 (regulation of fibroblast proliferation); GO:0048146 (positive regulation of fibroblast proliferation); GO:0048147 (negative regulation of fibroblast proliferation)
Cell Migration	GO:0016477 (cell migration); GO:0048870 (cell motility); GO:0002042 (cell migration involved in sprouting angiogenesis); GO:0044319 (wound healing, spreading of cells); GO:0035313 (wound healing, spreading of epidermal cells); GO:1903689 (regulation of wound healing, spreading of epidermal cells); GO:1903691 (positive regulation of wound healing, spreading of epidermal cells); GO:1903690 (negative regulation of wound healing, spreading of epidermal cells); GO:0040039 (inductive cell migration); GO:0050900 (leukocyte migration); GO:0030595 (leukocyte chemotaxis); GO:0002685 (regulation of leukocyte migration); GO:0002523 (leukocyte migration involved in inflammatory response); GO:0035701 (hematopoietic stem cell migration); GO:2000471 (regulation of hematopoietic stem cell migration); GO:2000473 (positive regulation of hematopoietic stem cell migration); GO:2000472 (negative regulation of hematopoietic stem cell migration); GO:0038089 (positive regulation of cell migration by vascular endothelial growth factor signaling pathway); GO:0038033 (positive regulation of endothelial cell chemotaxis by VEGF-activated vascular endothelial growth factor receptor signaling pathway); GO:0038090 (positive regulation of cell migration by VEGF-activated platelet derived growth factor receptor signaling pathway); GO:0030335 (positive regulation of cell migration); GO:1904849 (positive regulation of cell chemotaxis to fibroblast growth factor); GO:0010595 (positive regulation of endothelial cell migration); GO:1904859 (positive regulation of endothelial cell chemotaxis to vascular endothelial growth factor); GO:0010634 (positive regulation of epithelial cell migration); GO:0010763 (positive regulation of fibroblast migration); GO:1905212 (positive regulation of fibroblast chemotaxis); GO:0043542 (endothelial cell migration); GO:0043534 (blood vessel endothelial cell migration); GO:0035767 (endothelial cell chemotaxis); GO:0010594 (regulation of endothelial cell migration); GO:0060326 (cell chemotaxis); GO:1990956 (fibroblast chemotaxis); GO:0035441 (cell migration involved in vasculogenesis)
Tissue Remodeling	GO:0048771 (tissue remodeling; GO:0034103 (regulation of tissue remodeling); GO:0002248 (connective tissue replacement involved in inflammatory response wound healing); GO:1904596 (regulation of connective tissue replacement involved in inflammatory response wound healing); GO:0001974 (blood vessel remodeling); GO:0060312 (regulation of blood vessel remodeling); GO:0060313 (negative regulation of blood vessel remodeling); GO:2000504 (positive regulation of blood vessel remodeling); GO:0034105 (positive regulation of tissue remodeling); GO:0034104 (negative regulation of tissue remodeling); GO:1905205 (positive regulation of connective tissue replacement); GO:1905204 (negative regulation of connective tissue replacement); GO:0034102 (erythrocyte clearance); GO:0034106 (regulation of erythrocyte clearance); GO:0034107 (negative regulation of erythrocyte clearance); GO:0034108 (positive regulation of erythrocyte clearance); GO:0097709 (connective tissue replacement); GO:1905204 (negative regulation of connective tissue replacement); GO:1905205 (positive regulation of connective tissue replacement); GO:0002247 (clearance of damaged tissue involved in inflammatory response wound healing); GO:1904598 (positive regulation of connective tissue replacement involved in inflammatory response wound healing); GO:1904597 (negative regulation of connective tissue replacement involved in inflammatory response wound healing)
Epithelialization	GO:0043616 (keratinocyte proliferation); GO:0010837 (regulation of keratinocyte proliferation); GO:0010838 (positive regulation of keratinocyte proliferation); GO:0010839 (negative regulation of keratinocyte proliferation); GO:0050678 (regulation of epithelial cell proliferation); GO:0050680 (negative regulation of epithelial cell proliferation); GO:0050679 (positive regulation of epithelial cell proliferation); GO:0050678 (regulation of epithelial cell proliferation); GO:0050673 (epithelial cell proliferation)
Anti-Inflammatory Response	GO:0050728 (negative regulation of inflammatory response); GO:0002674 (negative regulation of acute inflammatory response); GO:0106015 (negative regulation of inflammatory response to wounding)
Antioxidation Activity	GO:0016209 (antioxidant activity); GO:0098869 (cellular oxidant detoxification); GO:0019430 (removal of superoxide radicals); GO:1904832 (negative regulation of removal of superoxide radicals); GO:1904833 (positive regulation of removal of superoxide radicals); GO:2000121 (regulation of removal of superoxide radicals); GO:0061692 (cellular detoxification of hydrogen peroxide); GO:0004601 (peroxidase activity); GO:0050605 (superoxide reductase activity); GO:0004784 (superoxide dismutase activity); GO:0032542 (sulfiredoxin activity); GO:0004791 (thioredoxin-disulfide reductase (NADPH) activity); GO:0045174 (glutathione dehydrogenase (ascorbate) activity); GO:0004362 (glutathione-disulfide reductase (NADPH) activity)
Detoxification	GO:1990748 (cellular detoxification); GO:0098754 (detoxification); GO:0097237 (cellular response to toxic substance); GO:0110052 (toxic metabolite repair); GO:0070458 (cellular detoxification of nitrogen compound)
Innate Antimicrobial Immune Response	GO:0045087 (innate immune response); GO:0140546 (defense response to symbiont); GO:0098542 (defense response to other organism); GO:0051707 (response to other organism); GO:0043207 (response to external biotic stimulus); GO:0006952 (defense response); GO:0042742 (defense response to bacterium); GO:0050832 (defense response to fungus); GO:0140367 (antibacterial innate immune response); GO:1900425 (negative regulation of defense response to bacterium); GO:1900426 (positive regulation of defense response to bacterium); GO:0050830 (defense response to Gram-positive bacterium); GO:0070944 (neutrophil-mediated killing of bacterium); GO:0050829 (defense response to Gram-negative bacterium); GO:1900424 (regulation of defense response to bacterium); GO:0070947 (neutrophil-mediated killing of fungus); GO:0061760 (antifungal innate immune response); GO:1900150 (regulation of defense response to fungus); GO:0140976 (host defense response against symbiont-mediated perturbation of plasma membrane integrity); GO:0034050 (symbiont-induced defense-related programmed cell death)

Notably, nearly one-third (32.52%) of the proteome was classified into processes involved in tissue remodeling and repair, including angiogenesis, epithelial migration, matrix remodeling, and immune modulation. This substantial representation suggests that FT and ACA preserve a diverse range of biomolecules that may help establish a local environment favorable to tissue support. This analysis builds on prior work, as CAMPs have remained largely unexplored in global proteome profiling. Earlier studies were limited to subsets of growth factors and selected biomolecules [9,13,31], whereas the current global proteomic profiling provides a more comprehensive view of the proteins retained in multilayer CAMPs.

## Data Availability

The data presented in this study are available on request from the corresponding author.

## Abbreviations

The following abbreviations are used in this manuscript:

AATB	American Association of Tissue Banks
ACA	Amnion–Intermediate–Chorion–Amnion
ANG-2	Angiopoietin-2
ANOVA	Analysis of Variance
BCA	Bicinchoninic Acid
BH	Benjamini–Hochberg
CAMPs	Cellular, Acellular, Matrix-like Products
Da	Dalton
ddaPASEF	Data-Dependent Acquisition–Parallel Accumulation-Serial Fragmentation
DPBS	Dulbecco’s Phosphate-Buffered Saline
ECM	Extracellular Matrix
EDTA	Ethylenediaminetetraacetic acid
EGF	Epidermal Growth Factor
ELISA	Enzyme-Linked Immunosorbent Assay
FC	Fold Change
FDA	Food and Drug Administration
FDR	False Discovery Rate
FT	Full-Thickness
GO	Gene Ontology
GOA	Gene Ontology Annotation
H&E	Hematoxylin and Eosin
HUVEC	Human Umbilical Vein Endothelial Cell
ISO	International Organization for Standardization
KEGG	Kyoto Encyclopedia of Genes and Genomes
KOG	EuKaryotic Orthologous Groups
L/min	Liter per Minutes
LC-MS/MS	Liquid Chromatography–Tandem Mass Spectrometry
LVES	Large Vessel Endothelial Supplement
M	Molar
m/z	mass-to-charge ratio
mM	microMolar
ms	Milliseconds
MTS	Masson’s Trichrome
ns	Not Significant
PDGF	Platelet-Derived Growth Factor-AA
PMSF	phenylmethylsulfonyl fluoride
RFU	Relative Fluorescence Unit
RPM	Rotations Per Minute
timsTOF	Trapped Ion Mobility Spectrometry-Time of Flight
µg/mL	Micrograms per Milliliter
µL	microLiter
µm	micrometer
V	Volts
Vs/cm2	Volts-seconds per square centimeter
VEGF	Vascular Endothelial Growth Factor
UHPLC	Ultra-High Performance Liquid Chromatography

## Appendix A

**Table A1:** Table of multiplex ELISA targets to Gene Ontology (GO) biological processes associated with tissue remodeling. Proteins from the plex ELISA panel were matched to GO terms associated with tissue remodeling and repair, including processes such as cell migration, angiogenesis, and inflammation. The proteins detected are categorized based on GO annotations detailed in Table 1.

Mechanisms	Proteins Detected
Wound Repair	Angiopoietin-1, Angiopoietin-2, Angiopoietin-4, ANGPTL1, ANGPTL2, ANGPTL7, Axl, WISP-1, CD40, Fractalkine, Endothelin, Endoglin, ErbB2, ErbB3, Epiregulin, FACX, F3, FGF-10, FGF Basic, Hepassocin, Frizzled-6, Frizzled-7, HB-EGF, IGF-I, IL-1 alpha, IL-6, Insulin, VEGF R2, Kininostatin, Lck, MMP-12, HRG-alpha, PDGF-AA, PDGF-BB, PDGF R alpha, PECAM-1, PF4, uPA, uPAR, Angiostatin, SAA, Shh-N, TFPI, TGF-beta 1, TGF-beta 2, TGF-beta RI, Thrombospondin-1, TIMP-1, TLR4, TMEFF2, Dtk, VEGF, VEGF-B
Angiogenesis	Activin RIA, Angiogenin, Angiopoietin-1, Angiopoietin-2, Angiopoietin-4, Amphiregulin, Bax, BMP-2, BMP-4, BMP-5, BMP-7, CV-2, BMPR-IA, BMPR-II, Eotaxin, MCP-1, Eotaxin-2, CTGF, NOV, CCR2, CCR3, CD40, VE-Cadherin, Cerberus 1, Cryptic, Endostatin, Cripto-1, M-CSF, M-CSF R, G-CSF R, beta-Catenin, Fractalkine, IP-10, BLC, IL-8, CXCR3, Decorin, Dkk-1, EDA-A2, EDAR, Endothelin, EGF, EGF R, Endoglin, ErbB2, Epiregulin, Endocan, EYA2, F3, Fas Ligand, DANCE, FGF-10, FGF-16, FGF-18, FGF Basic, FGF-6, FGF-7, FGF-8, FGF-9, VEGF R3, Frizzled-3, Frizzled-4, Frizzled-5, Frizzled-6, GDF11, GDF3, GDNF, Glypican 3, Osteoactivin, Grb2, GREMLIN, Progranulin, IGF-I, IGFBP-rp1, IL-10, IL-17F, IL-1 alpha, IL-1 beta, IL-6, sgp130, Activin A, Activin B, VEGF R2, Leptin, Leptin R, LIF, LRP-1, LRP-6, HGFR, MFG-E8, MMP-12, MMP-14, MMP-15, MMP-19, MMP-2, MMP-20, MMP-8, MMP-9, NGF R, NrCam, NRG3, Neuropilin-2, NT-4, PDGF-AA, PDGF-BB, PDGF-C, PDGF R alpha, PDGF R beta, PECAM-1, PF4, PlGF, Angiostatin, Prolactin, EG-VEGF, RELT, ROBO4, EDG-1, sFRP-1, Shh-N, Smad 1, Smad 4, Smad 5, Smad 7, SPARC, TGF-alpha, TGF-beta 1, TGF-beta 2, TGF-beta 3, TGF-beta RI, TGF-beta RII, TGF-beta RIII, Thrombospondin-1, Thrombospondin-2, Thrombospondin-4, Tie-1, TLR3, TWEAK R, TWEAK, PD-ECGF, VEGF, VEGF-B, VEGF-C, VEGF-D
Inflammatory Response	D6, Activin RIA, Angiopoietin-1, Angiopoietin-2, Angiopoietin-4, ANGPTL1, ANGPTL2, ANGPTL7, Axl, Bax, BMP-2, BMP-6, BMPR-IB, I-309, Eotaxin, MCP-4, CCL14, MIP-1d, HCC-4, Tarc, PARC, MIP-3 beta, MCP-1, MIP-3 alpha, MDC, Eotaxin-2, TECK, Eotaxin-3, CTACK, CCL28, MIP-1a, MIP-1b, RANTES, MCP-3, MCP-2, CTGF, WISP-1, CCR1, CCR2, CCR3, CCR4, CCR5, CCR6, CCR7, HCR, CD14, CD 163, CD40, CNTF, M-CSF, M-CSF R, Fractalkine, GRO-a, IP-10, I-TAC, BLC, MIP 2, GRO, ENA-78, GCP-2, IL-8, MIG, CXCR2, CXCR3, CXCR4, CXCR6, Endothelin, Endoglin, Erythropoietin, ErbB2, ErbB3, Epiregulin, FACX, F3, FGF-10, FGF Basic, FGF-4, FGF-7, Hepassocin, Frizzled-6, Frizzled-7, Progranulin, Granzyme A, HB-EGF, IFN-gamma, IFN-gamma R1, IGF-I, IL-10, IL-10 R beta, IL-13, IL-17, IL-17B, IL-17C, IL-17D, IL-17F, IL-17R, IL-17RC, IL-18 R alpha, IL-18 R beta, IL-1 alpha, IL-1 beta, IL-1 F10, IL-1 RI, IL-1 R3, IL-1 R4, IL-1 R6, IL-1 ra, IL-20 R beta, IL-22, IL-23, IL-23 R, IL-17E, IL-27, IL-2 R alpha, IL-31 RA, IL-1 F6, IL-1 F8, IL-1 F9, IL-1 F7, IL-4, IL-4 R, IL-5, IL-5 R alpha, IL-6, IL-6 R, IL-9, Activin A, Activin B, Insulin, LFA-1 alpha, MAC-1, VEGF R2, Kininostatin, Kremen-1, LBP, Lck, Lipocalin-2, LRP-1, MIF, MMP-12, MMP-2, MMP-25, HRG-alpha, PDGF-AA, PDGF-BB, PDGF R alpha, PECAM-1, PF4, uPA, uPAR, Angiostatin, Pentraxin3, EN-RAGE, SAA, E-Selectin, P-selectin, Shh-N, SIGIRR, Smad 1, TFPI, TGF-beta 1, TGF-beta 2, TGF-beta RI, Thrombospondin-1, TIMP-1, TLR1, TLR2, TLR3, TLR4, TMEFF2, TSG-6, TWEAK R, TNF RI, TNF RII, OX40 Ligand, Dtk, VCAM-1, VEGF, VEGF-B, Lymphotactin
Proliferation	Activin RIIA, Adiponectin, Angiogenin, Angiopoietin-1, Angiopoietin-4, Amphiregulin, Artemin, Bax, BMP-2, BMP-4, BMP-5, BMP-6, BMP-7, CV-2, BMPR-IA, BMPR-IB, BMPR-II, BTC, Eotaxin, CCL14, MIP-3 beta, MCP-1, Eotaxin-2, Eotaxin-3, RANTES, MCP-2, CTGF, NOV, WISP-1, CCR2, CCR3, CD40, B7-1, VE-Cadherin, Cerberus 1, CLC, CNTF, CNTF R alpha, Endostatin, Cripto-1, TSLP R, M-CSF, M-CSF R, GM-CSF, GM-CSF R alpha, GCSF, G-CSF R, Csk, Cardiotrophin-1, CTLA-4, beta-Catenin, Fractalkine, GRO-a, IP-10, I-TAC, SDF-1, ENA-78, IL-8, MIG, CXCR2, CXCR3, Endothelin, EGF, EGF R, Endoglin, Erythropoietin, ErbB2, ErbB3, ErbB4, Epiregulin, Endocan, F3, FADD, Fas Ligand, DANCE, FGF-10, FGF-13 1B, FGF-16, FGF-17, FGF-18, FGF-19, FGF Basic, FGF-20, FGF-21, FGF-23, FGF-4, FGF-5, FGF-6, FGF-7, FGF-8, FGF-9, FGF-BP, FGF R3, FGF R4, FGF R5, Hepassocin, VEGF R3, Follistatin, Frizzled-3, Frizzled-4, Frizzled-5, Frizzled-6, Frizzled-7, GDF11, GDF5, GDF9, GDNF, GHR, Glypican 3, Osteoactivin, GREMLIN, Progranulin, HB-EGF, HGF, IFN-beta, IFN-gamma, IGF-I, IGF-I R, IGF-II, IGFBP-2, IGFBP-3, IGFBP-4, IGFBP-6, IGFBP-rp1, IL-10, IL-11, IL-12 p70, IL-12 p40, IL-12 R beta 1, IL-12 R beta 2, IL-13, IL-13 R alpha 2, IL-15, IL-17, IL-18 R beta, IL-1 alpha, IL-1 beta, IL-2, IL-20 R beta, IL-21, IL-23, IL-23 R, IL-24, IL-26, IL-27, IL-2 R alpha, IL-3, IL-31 RA, IL-3 R alpha, IL-4, IL-5, IL-5 R alpha, IL-6, IL-6 R, sgp130, IL-7, IL-7 R alpha, IL-9, Activin A, Insulin, Insulin R, VEGF R2, SCF, Leptin, Leptin R, Galectin-3, LIF, LIF R alpha, MIF, MMP-12, MMP-14, MMP-16, MMP-2, MMP-7, MMP-9, beta-NGF, NGF R, HRG-alpha, Neuropilin-2, NT-3, OSM, Osteocrin, PDGF-AA, PDGF-BB, PDGF-C, PDGF-D, PDGF R alpha, PDGF R beta, PF4, PlGF, uPA, Angiostatin, Prolactin, EG-VEGF, RELM beta, EDG-1, sFRP-1, sFRP-4, Shh-N, SLPI, Smad 1, Smad 4, Smad 7, SPARC, TGF-alpha, TGF-beta 1, TGF-beta 2, TGF-beta 3, TGF-beta RI, TGF-beta RII, TGF-beta RIII, Thrombospondin-1, Thrombospondin-4, Thrombopoietin, Tie-1, TIMP-1, TLR4, HVEM, TNF RII, DR6, TRANCE, TWEAK, OX40 Ligand, VCAM-1, VEGF, VEGF-B, VEGF-C, VEGF-D, Lymphotactin
Cell Migration	D6, Activin RIA, Activin RIB, Angiogenin, Angiopoietin-1, Angiopoietin-2, Angiopoietin-4, Artemin, Axl, Bax, BMP-2, BMP-4, BMP-5, BMP-7, CV-2, BMPR-II, I-309, Eotaxin, MCP-4, CCL14, MIP-1d, HCC-4, Tarc, PARC, MIP-3 beta, MCP-1, MIP-3 alpha, MDC, Eotaxin-2, TECK, Eotaxin-3, CTACK, CCL28, MIP-1a, MIP-1b, RANTES, MCP-3, MCP-2, CTGF, NOV, WISP-1, CCR1, CCR2, CCR3, CCR4, CCR5, CCR6, CCR7, CCR8, CCR9, HCR, CD40, VE-Cadherin, Cerberus 1, Cripto-1, M-CSF, M-CSF R, GM-CSF, G-CSF R, beta-Catenin, Fractalkine, GRO-a, IP-10, I-TAC, SDF-1, BLC, CXCL14, CXCL16, MIP 2, GRO, ENA-78, GCP-2, IL-8, MIG, CXCR1, CXCR2, CXCR3, CXCR4, CXCR5, CXCR6, Decorin, Endothelin, EGF, EGF R, Endoglin, ErbB4, FACX, F3, FADD, FGF-10, FGF-13 1B, FGF-16, FGF-18, FGF-19, FGF Basic, FGF-4, FGF-7, FGF-8, FGF-9, FGF-BP, FGF R4, VEGF R3, Follistatin-like 1, Frizzled-3, Frizzled-4, GDF-15, GDNF, GFR alpha-1, GFR alpha-3, Glypican 3, Glypican 5, Osteoactivin, GREMLIN, Progranulin, HB-EGF, HGF, ICAM-1, IFN-gamma, IGF-I, IGF-I R, IGFBP-6, IL-10, IL-12 p70, IL-12 p40, IL-16, IL-17, IL-17R, IL-17RC, IL-1 beta, IL-1 RI, IL-23, TCCR, IL-4, IL-6, IL-6 R, Insulin, Insulin R, LFA-1 alpha, VEGF R2, SCF, LBP, Lck, Lipocalin-2, Leptin, Galectin-3, HGFR, MIF, MMP-12, MMP-14, MMP-2, MMP-7, MMP-9, DAN, NrCam, NRG3, Neuropilin-2, Neurturin, NT-3, PDGF-AA, PDGF-BB, PDGF-C, PDGF-D, PDGF R alpha, PDGF R beta, PECAM-1, PF4, PlGF, uPA, Angiostatin, ROBO4, EN-RAGE, EDG-1, SAA, E-Selectin, L-Selectin, P-selectin, Shh-N, Smad 4, SPARC, TGF-beta 1, TGF-beta 2, TGF-beta RI, TGF-beta RII, TGF-beta RIII, Thrombospondin-1, Thrombospondin-4, TLR4, TMEFF2, TSG-6, TWEAK R, HVEM, TRANCE, TWEAK, TRADD, TREM-1, Dtk, VCAM-1, VEGF, VEGF-B, VEGF-C, VEGF-D, Lymphotactin
Tissue Remodeling	Axl, Bax, BMPR-II, CCR2, M-CSF R, Csk, beta-Catenin, FGF-10, FGF-8, VEGF R3, GDF5, Osteoactivin, GREMLIN, IGF-I, IL-12 p40, IL-15, IL-1 alpha, IL-2, IL-20 R alpha, IL-21, IL-23, IL-6, IL-7, Leptin, Leptin R, LIF, MMP-14, MMP-2, Angiostatin, EDG-1, sFRP-1, TGF-beta 1, TGF-beta 2, Thrombospondin-4, Tie-1, TIMP-1, TRANCE
Epithelialization	sFRP-1, GDF5, MMP-12, Eotaxin-2, SPARC, MCP-1, IL-12 p40, ErbB4, Tie-1, Endoglin, Thrombospondin-1, TGF-beta RIII, Frizzled-7, Eotaxin-3, VEGF-C, VEGF-B, IL-10, CXCR3, IL-26, TGF-beta 2, SDF-1, Angiogenin, F3, Thrombospondin-4, IL-12 p70, Prolactin, PDGF-BB, NGF R, ErbB3, IGF-II, Neuropilin-2, FGF-18, FGF-16, VEGF R3, TWEAK, BMP-6, BMP-5, FGF-BP, Angiopoietin-1, ErbB2, CCR3, Eotaxin, Leptin, BMPR-IA, RANTES, RELM beta, HGF, EGF R, Glypican 3, TGF-beta 1, EGF, Progranulin, VEGF R2, Shh-N, Epiregulin, IGFBP-3, TRANCE, TGF-beta RI, Hepassocin, IGFBP-4, FGF Basic, EG-VEGF, FGF-10, FGF-7, Angiopoietin-4, Activin RIIA, IL-17, IGF-I, CV-2, MMP-14, beta-Catenin, BMP-4, Bax, Follistatin, TGF-alpha, NOV, Amphiregulin, VEGF, IL-6, FGF-9, BMPR-II
Innate Immune Response	B7-1, MMP-12, GCSF, MMP-8, Adiponectin, IL-24, IL-10 R alpha, FGF-10, Frizzled-5, GM-CSF, IL-18 BPa, FGF-19, Glypican 3, Bax, VEGF-D, IL-12 R beta 2, MMP-9, G-CSF R, Activin A, Activin B, IL-17B R, MCP-3, MCP-2, TMEFF1, CNTF, Smad 1, CCR5, CCR6, MDC, IL-1 F10, D6, CXCR6, E-Selectin, TRAIL R2, GRO-a, IL-1 R4, IL-10 R beta, M-CSF, GRO, MIP 2, Tarc, Pentraxin3, Eotaxin-2, PF4, IL-17C, RANTES, MCP-1, IL-4 R, VCAM-1, IL-5 R alpha, TLR1, MMP-3, Fractalkine, CXCR4, HCC-4, TECK, IL-18 R alpha, MIF, IL-22, MIP-1b, FADD, IP-10, CCL28, Galectin-3, TSG-6, MMP-25, Thrombospondin-1, HCR, Insulin, IL-1 F9, IL-1 F8, IL-1 F7, IL-27, CXCL14, IL-18 R beta, Eotaxin-3, TNF RII, Axl, IFN-alpha/beta R2, CTACK, CCR2, I-309, ENA-78, CXCR3, CXCL16, SAA, IL-17B, IL-31 RA, Lymphotactin, IL-1 F6, IL-8, LFA-1 alpha, IFN-alpha/beta R1, MIP-1d, CCL14, IL-1 R3, SDF-1, Fas Ligand, IFN-gamma R1, IL-9,Cripto-1, OX40 Ligand, I-TAC, M-CSF R, F3, IL-13, LRP-1, TIMP-1, BMP-2, IL-17D, IL-17E, Granzyme A, CXCR2, PARC, CD14, MIG, IL-1 ra, MAC-1, BMPR-IB, Kininostatin, IL-2 R alpha, Erythropoietin, IL-1 alpha, IFN-gamma, IFN-beta, IL-5, IL-4, SIGIRR, MIP-1a, CD40, Activin RIA, Grb2, Endothelin, BMP-6, CCR7, CCR1, IL-12 R beta 1, MCP-4, MIP-3 beta, IL-20 R beta, CCR9, CCR4, CCR3, Eotaxin, IL-1 RI, IL-21, CD 163, IL-1 R6, TNF RI, TLR3, IL-10, BLC, MICA, MIP-3 alpha, Lipocalin-2, SLPI, EN-RAGE, IL-17R, IL-17RC, TGF-beta 1, GCP-2, IL-7 R alpha, TCCR, Angiogenin, TLR2, IL-12 p70, IL-1 beta, IL-22 R, TLR4, HVEM, MMP-7, DEFA5, P-selectin, IL-12 p40, BD-1, IL-23, IL-17F, IL-17, IL-6 R, LBP, IL-23 R, IL-6, TREM-1, Progranulin
